# Clinical Profile of Venous Thromboembolism and Applicability of the ThroLy and IMPEDE VTE Risk Scores in Patients With Lymphoma and Multiple Myeloma: A Moroccan Single-Center Retrospective Study

**DOI:** 10.7759/cureus.113710

**Published:** 2026-07-31

**Authors:** Oumayma Mnafe, Asmae Jamlaoui, Hicham Eddou

**Affiliations:** 1 Hematology and Medical Oncology, Hôpital Militaire Moulay Ismail, Meknes, MAR

**Keywords:** hodgkin's lymphoma, impede vte score, multiple myeloma treatment, non hodgkin's lymphoma, throly score, thrombosis

## Abstract

Background

Venous thromboembolism (VTE), encompassing deep vein thrombosis (DVT) and pulmonary embolism (PE), is a major complication in patients with hematologic malignancies and contributes substantially to morbidity and mortality. Patients with lymphoma and multiple myeloma are particularly vulnerable because of disease-related and treatment-related prothrombotic factors. Risk assessment models, including the Thrombosis in Lymphoma (ThroLy) score and the IMPEDE VTE score, have been developed to identify patients at increased thrombotic risk. This study aimed to describe the clinical characteristics of thromboembolic events in patients with lymphoma and multiple myeloma and to evaluate the clinical applicability of these risk assessment tools in a Moroccan cohort.

Methods

We conducted a retrospective, descriptive, and analytical study in the Department of Clinical Hematology at Moulay Ismail Military Hospital, Meknes, Morocco, between September 2023 and September 2025. A total of 141 patients were included, comprising 72 patients with lymphoma and 69 patients with multiple myeloma. Clinical, demographic, treatment-related, and thrombotic data were collected from medical records. Thrombotic risk was assessed at diagnosis using the ThroLy score in patients with lymphoma and the IMPEDE VTE score in patients with multiple myeloma.

Results

Among the 141 patients included, 22 patients developed VTE, accounting for a total of 24 thrombotic events, as two patients experienced recurrent VTE during follow-up. The mean age of patients with thrombosis was 51 years, and 15 of the 22 patients (68.2%) were men. Proximal DVT represented 23 of 24 thrombotic events (95.8%), while PE was documented in 2 of 24 events (8.3%). Central venous catheterization was the most frequently identified associated factor, observed in eight patients (36.4%), followed by corticosteroid therapy in four patients (22.2%) and erythropoietin exposure in two patients (11.1%). In the lymphoma cohort, a high ThroLy score was significantly associated with thrombosis (p < 0.001). In the multiple myeloma cohort, 85.7% of patients who developed thrombosis were classified as high risk according to the IMPEDE VTE score.

Conclusions

VTE remains a frequent complication among patients with hematologic malignancies. The ThroLy and IMPEDE VTE scores demonstrated clinical utility for identifying patients at increased thrombotic risk. Their systematic implementation may improve risk stratification and support personalized thromboprophylaxis strategies in routine hematology practice.

## Introduction

Venous thromboembolism (VTE), including deep vein thrombosis (DVT) and pulmonary embolism (PE), is a major cause of morbidity and mortality in patients with cancer. Compared with the general population, patients with malignancies have a four- to seven-fold increased risk of developing thromboembolic complications. This risk is particularly pronounced in hematologic malignancies because of the combined effects of cancer-related hypercoagulability, endothelial dysfunction, inflammatory activation, treatment-related factors, and patient-related comorbidities [1].

Among hematologic cancers, lymphoma and multiple myeloma are associated with a particularly high incidence of VTE. In patients with lymphoma, advanced-stage disease, bulky tumors, hospitalization, reduced mobility, central venous catheterization, and chemotherapy exposure have been identified as major contributors to thrombogenesis [2,3]. In multiple myeloma, the risk is further increased by disease-related factors and by the use of immunomodulatory drugs (IMiDs), such as thalidomide and lenalidomide, especially when combined with high-dose corticosteroids or cytotoxic chemotherapy.

Several disease-specific predictive models have been developed for this purpose. The Thrombosis in Lymphoma (ThroLy) score was designed to estimate the risk of VTE in patients with lymphoma based on clinical and laboratory parameters [2]. Similarly, the IMPEDE VTE score was developed and validated to predict thrombotic risk in patients with multiple myeloma receiving contemporary treatment regimens [3]. These tools have demonstrated promising predictive performance in several international studies and are increasingly incorporated into routine clinical practice.

Despite the growing body of evidence regarding cancer-associated thrombosis, data from North African countries remain limited. Differences in demographic characteristics, disease presentation, treatment practices, and access to supportive care may influence both the incidence of thrombotic complications and the performance of predictive models. To date, few studies have specifically evaluated thrombotic risk assessment tools in Moroccan patients with hematologic malignancies.

Although the ThroLy and IMPEDE VTE scores have been developed and validated in predominantly European and North American populations, evidence regarding their applicability in North African patients remains scarce. Differences in genetic background, disease characteristics, treatment patterns, healthcare organization, and access to supportive care may influence the incidence of VTE and the performance of risk assessment models. To our knowledge, no previous Moroccan study has evaluated the clinical applicability of these disease-specific risk assessment tools in patients with lymphoma and multiple myeloma. Therefore, this study aimed to describe the clinical profile of VTE and assess the clinical applicability of the ThroLy and IMPEDE VTE scores in a real-world Moroccan cohort.

## Materials and methods

Study design and setting

This retrospective, descriptive, and analytical study was conducted in the Department of Clinical Hematology at Moulay Ismail Military Hospital, Meknes, Morocco. The study covered a two-year period extending from September 2023 to September 2025.

Study population

All consecutive adult patients diagnosed with lymphoma or multiple myeloma and followed in the department during the study period were screened for eligibility. Diagnoses were established according to standard clinical, histopathological, immunophenotypic, and radiological criteria.

Patients were included regardless of whether they developed a thrombotic event during follow-up. Patients with incomplete medical records or missing data preventing thrombotic risk assessment were excluded from the analysis. A total of 141 patients met the eligibility criteria and were included in the study, comprising 72 patients with lymphoma and 69 patients with multiple myeloma.

Data collection

Clinical data were collected retrospectively from medical records and hospital databases. The variables analyzed included demographic characteristics (age and sex), diagnosis, disease characteristics, treatment modalities, use of central venous catheters, exposure to corticosteroids and erythropoietin, occurrence and type of thrombotic events, anticoagulant management, and outcome data when available.

VTE was defined as objectively confirmed DVT and/or PE, diagnosed by Doppler ultrasonography, computed tomography pulmonary angiography, or other validated imaging modalities according to institutional practice.

Thrombotic risk assessment

At diagnosis, thrombotic risk was assessed using disease-specific predictive models.

Patients with lymphoma were evaluated using the ThroLy score, which incorporates several clinical and laboratory variables associated with thrombotic risk, including previous thrombosis, obesity, extranodal disease, mediastinal involvement, reduced mobility, and hematological abnormalities.

Patients with multiple myeloma were assessed using the IMPEDE VTE score, which integrates patient-related and treatment-related risk factors such as body mass index, previous VTE, fractures, erythropoietin exposure, dexamethasone use, IMiD therapy, and thromboprophylaxis status.

Patients were categorized into risk groups according to the original definitions and recommended thresholds of each scoring system.

Outcomes

The primary outcome was the occurrence of objectively confirmed VTE during the study period.

Secondary outcomes included characterization of thrombotic events according to their anatomical location, clinical presentation, associated risk factors, and anticoagulant management, as well as evaluation of the clinical applicability of the ThroLy and IMPEDE VTE scores.

Statistical analysis

Data were analyzed using descriptive and analytical statistical methods. Continuous variables were summarized using means and ranges, whereas categorical variables were expressed as frequencies (n) and percentages (%).

The incidence of thrombotic events was calculated for each disease subgroup. Associations between thrombotic risk categories and thrombosis occurrence were assessed using univariate analysis. Statistical significance was defined as a two-sided p-value of less than 0.05.

Ethical considerations

The study was conducted in accordance with the principles of the Declaration of Helsinki and institutional ethical standards. Patient confidentiality was maintained throughout the study, and all data were anonymized before analysis.

## Results

Baseline characteristics

A total of 141 patients were included in the study, including 72 patients with lymphoma and 69 patients with multiple myeloma. The mean age was 54 years in the lymphoma cohort and 62 years in the multiple myeloma cohort. Men predominated in both groups, with male-to-female ratios of 1.4 and 2.13, respectively. Disseminated disease was documented in 59 of 72 lymphoma patients (81.9%).

Thrombotic events occurred in 13 of 72 patients with lymphoma (18.1%) and in 9 of 69 patients with multiple myeloma (13.0%) (Table 1).

**Table 1:** Baseline Characteristics of the Study Cohort

Characteristic	Lymphoma (n = 72, 51.1%)	Multiple Myeloma (n = 69, 48.9%)
Mean age (years)	54	62
Male-to-female ratio	1.4	2.13
Disseminated disease, n (%)	59 (81.9%)	Not available
Thrombotic events, n (%)	13 (18.1%)	9 (13.0%)

Characteristics of thrombotic events

A total of 24 thrombotic events were recorded. The mean age at thrombosis was 51 years. Men accounted for 15 of 24 thrombotic events (62.5%). Proximal DVT represented the predominant presentation, occurring in 23 of 24 events (95.8%). PE was diagnosed in 2 of 24 events (8.3%). Most thrombotic events were symptomatic and clinically apparent at diagnosis (Table 2).

**Table 2:** Characteristics of Thrombotic Events (n = 24)

Variable	Value
Mean age at thrombosis (years)	51
Male sex	68.2% (15/22)
Proximal thrombosis	95.8% (23/24)
Pulmonary embolism	8.3% (2/24)
Symptomatic presentation	87.5% (21/24)

Central venous catheterization was the most frequently identified associated risk factor, observed in eight patients (36.4%). Corticosteroid therapy was documented in four patients (18.2%), while erythropoietin exposure was identified in two patients (9.1%) (Table 3).

**Table 3:** Associated Risk Factors in Patients With Thrombosis

Risk Factor	n (%)
Central venous catheter (port-a-cath)	8 (36.4%)
Corticosteroid therapy	4 (18.2%)
Erythropoietin use	2 (9.1%)

Clinical applicability of the ThroLy score

Among patients with lymphoma who developed thrombosis, 50% were classified as high risk according to the ThroLy score at diagnosis. Univariate analysis demonstrated a statistically significant association between a high-risk ThroLy category and thrombosis occurrence (p < 0.001) (Table 4).

**Table 4:** ThroLy Score Performance in the Lymphoma Cohort

Finding	Value
Patients with thrombosis classified as high risk	50% (6/12)
Association with thrombosis	p < 0.001

Clinical applicability of the IMPEDE VTE score

Among patients with multiple myeloma who developed thrombosis, six of seven evaluable patients (85.7%) were categorized as high risk according to the IMPEDE VTE score (Table 5).

**Table 5:** IMPEDE VTE Score Performance in the Multiple Myeloma Cohort

Finding	Value
Patients with thrombosis classified as high risk	85.7% (6/7)

## Discussion

This study provides one of the few descriptions of cancer-associated thrombosis in Moroccan patients with hematologic malignancies. The findings highlight the substantial burden of VTE in both lymphoma and multiple myeloma and support the clinical relevance of disease-specific risk assessment tools in routine hematology practice. Cancer-associated thrombosis remains a major cause of morbidity and mortality worldwide and represents a significant challenge in hematologic oncology [1-4].

The overall thrombotic burden observed in this cohort is consistent with the recognized prothrombotic nature of hematologic cancers. Lymphoma patients exhibited a thrombosis incidence of 18.1%, while multiple myeloma patients had an incidence of 13.0%. Although direct comparisons between studies are challenging because of differences in patient populations, treatment regimens, and methods of event ascertainment, these rates are within the range reported internationally for high-risk hematologic populations [5,6]. The predominance of proximal DVT is clinically important, as proximal events are associated with a greater risk of PE, recurrent thrombosis, and post-thrombotic syndrome than distal thrombosis [7,8].

A notable finding was the strong association between central venous catheter use and thrombosis. Nearly half of thrombotic events occurred in patients with a port-a-cath. Central venous access devices are indispensable for chemotherapy administration and supportive care, but they also create local endothelial injury and flow disturbances that promote thrombogenesis. Previous studies have consistently identified catheter-related thrombosis as an important complication in hematologic patients and cancer populations in general [9-11]. The present findings reinforce the need for meticulous catheter management, early recognition of catheter dysfunction, and individualized consideration of thromboprophylaxis in selected high-risk patients.

Corticosteroid exposure was another frequently associated factor. Corticosteroids are widely used in both lymphoma and multiple myeloma treatment protocols and have been implicated in increased thrombotic risk through multiple mechanisms, including endothelial activation, metabolic alterations, and interactions with other prothrombotic therapies. The observation that all myeloma patients with thrombosis were receiving IMiD-based therapy is particularly relevant. Immunomodulatory agents such as thalidomide and lenalidomide are well-established risk factors for VTE, especially when combined with corticosteroids or chemotherapy [4,7,12]. Current international guidelines from the American Society of Clinical Oncology (ASCO), the American Society of Hematology (ASH), the International Initiative on Thrombosis and Cancer (ITAC), and the National Comprehensive Cancer Network (NCCN) recommend risk-adapted thromboprophylaxis in patients receiving these agents [13-15].

The performance of the ThroLy score in this cohort is encouraging. A high ThroLy score was significantly associated with thrombosis, with a p-value < 0.001. The ThroLy model was originally developed and validated to predict VTE in lymphoma patients using clinical and laboratory variables such as previous thrombosis, obesity, mediastinal involvement, reduced mobility, extranodal disease, and hematological abnormalities [2]. Although external validation studies have shown variable performance across different populations, the score remains a useful tool for identifying patients at increased thrombotic risk [2]. Our findings support its applicability in a Moroccan clinical setting, although larger prospective studies are required to better evaluate its discrimination and calibration performance.

The IMPEDE VTE score also demonstrated clinical relevance. The majority of myeloma patients who developed thrombosis were categorized as high risk at diagnosis. This score incorporates body mass index, previous VTE, fractures, erythropoietin exposure, dexamethasone use, IMiD therapy, and other patient-related variables [3]. Since its development, several validation studies have confirmed its ability to stratify patients into distinct thrombotic risk categories and to outperform older consensus-based approaches [3,7]. The present results are consistent with these observations and suggest that IMPEDE VTE may facilitate the identification of myeloma patients who could benefit from intensified thromboprophylaxis strategies.

Recent studies evaluating primary thromboprophylaxis in cancer patients have demonstrated that direct oral anticoagulants may reduce the incidence of VTE in selected high-risk populations without substantially increasing major bleeding events [10]. Consequently, accurate risk stratification using validated predictive models has become increasingly important to optimize the balance between thrombosis prevention and bleeding risk [4,6].

Several limitations should be acknowledged. First, the study was retrospective and monocentric, which introduces the possibility of selection bias and incomplete documentation. Second, the sample size was relatively small, particularly for subgroup analyses. Third, multivariable modeling, receiver operating characteristic analysis, and formal estimates of sensitivity and specificity were not available. Fourth, the study did not systematically capture bleeding outcomes, which are essential when evaluating prophylactic strategies [4,6]. Finally, the absence of long-term follow-up limits assessment of recurrent thrombosis and survival outcomes.

Despite these limitations, the study has notable strengths. It represents real-world practice in a North African hematology center, includes both lymphoma and myeloma populations, and evaluates two disease-specific risk models in the same institutional setting. Given the scarcity of regional data, these findings contribute to the understanding of cancer-associated thrombosis in Moroccan patients and support the integration of structured thrombotic risk assessment into routine hematology practice [1,5,6]. Future prospective multicenter studies are needed to validate these results and to determine the optimal thromboprophylaxis strategies for patients with hematologic malignancies.

## Conclusions

VTE remains a significant complication in patients with lymphoma and multiple myeloma. In this Moroccan single-center cohort, thrombotic events were predominantly proximal and symptomatic, and central venous catheterization emerged as a major associated factor. The ThroLy score showed a significant association with ThroLy patients, while the IMPEDE VTE score effectively identified high-risk myeloma patients. Systematic use of these validated risk assessment tools may improve early risk stratification and support more personalized thromboprophylaxis strategies in hematologic malignancies.
